# Beyond endocrine resistance: estrogen receptor (ESR1) activating mutations mediate chemotherapy resistance through the JNK/c-Jun MDR1 pathway in breast cancer

**DOI:** 10.1007/s10549-024-07507-3

**Published:** 2024-10-29

**Authors:** Marwa Taya, Keren Merenbakh-Lamin, Asia Zubkov, Zohar Honig, Alina Kurolap, Ori Mayer, Noam Shomron, Ido Wolf, Tami Rubinek

**Affiliations:** 1 Department of Oncology, Tel Aviv Sourasky Medical Center, 6 Weizmann St., 6423906 Tel Aviv, Israel; 2 The Faculty of Medical and Health Sciences, Tel Aviv University, Tel Aviv, Israel; 3 Institute of Pathology, Tel Aviv Sourasky Medical Center, Tel Aviv, Israel; 4 The Genetics Institute and Genomics Center, Tel Aviv Sourasky Medical Center, Tel Aviv, Israel

**Keywords:** Chemoresistance, Activating mutations, MDR1, JNK/c-Jun signaling pathway, JNK inhibitor

## Abstract

**Purpose:**

All patients with metastatic breast cancer (MBC) expressing estrogen receptor-α (ESR1) will eventually develop resistance to endocrine therapies. In up to 40% of patients, this resistance is caused by activating mutations in the ligand-binding domain (LBD) of ESR1. Accumulating clinical evidence indicate adverse outcomes for these patients, beyond that expected by resistance to endocrine therapy. Here we aimed to study the role of ESR1 mutations in conferring chemoresistance in BC cells.

**Methods:**

MCF-7 cells harboring Y537S and D538G ESR1 mutations (mut-ER) were employed to study the response to chemotherapy drugs, paclitaxel and doxorubicin, using viability and apoptotic assay in vitro, and tumor growth in vivo. JNK/c-Jun/MDR1 pathway was studied using qRT-PCR, western-blot, gene-reporter and ChIP assays. *MDR1* expression was analyzed in clinical samples using IHC.

**Results:**

Cell harboring ESR1 mutations displayed relative chemoresistance compared to WT-ER, evidenced by higher viability and reduced apoptosis as well as resistance to paclitaxel in vivo. To elucidate the underlying mechanism, *MDR1* expression was examined and elevated levels were observed in mut-ER cells, and in clinical BC samples. *MDR1* is regulated by the c-Jun pathway, and we showed high correlation between these two genes in BC using TCGA databases. Accordingly, we detected higher JNK/c-Jun expression and activity in ESR1-mutated cells, as well as increased occupancy of c-Jun in *MDR1* promoter. Importantly, JNK inhibition decreased *MDR1* expression and restored sensitivity to chemotherapy.

**Conclusions:**

Taken together, these data indicate that *ESR1* mutations confer chemoresistance through activation of the JNK/MDR1 axis. These finding suggest a novel treatment option for BC tumors expressing ESR1 mutations.

**Supplementary Information:**

The online version contains supplementary material available at 10.1007/s10549-024-07507-3.

## Introduction

Breast cancer (BC) is the most frequently diagnosed malignancy and the leading cause of cancer related death among women [1]. About 75% of patients with BC express estrogen receptor-α (*ESR1*) and endocrine therapy is the mainstay treatment for these patients. While being effective and safe, some patients with metastatic breast cancer (MBC) do not respond to any form of endocrine treatment (de novo resistance), and virtually all patients who initially respond will eventually develop endocrine resistance (acquired resistance) [2, 3]. We and others have discovered activating mutations in the ligand-binding domain (LBD) of the *ESR1* as an acquired mechanism for endocrine resistance [2–6], with D538G and Y537S being the most common mutations [7–9]. These mutations arise following endocrine treatment, most commonly aromatase inhibitors, and are identified in up to 40% of patients with ER(α)-positive MBC [7, 8, 10].

Accumulating clinical and laboratory data suggest a unique aggressive phenotype of BC expressing these mutations beyond merely endocrine resistance. Thus, we and others have shown predilection of tumors and cells harboring these mutations to form liver metastasis [2, 11, 12]. Moreover, these data indicates that following the development of endocrine resistance, the outcomes for patients with tumors harboring ESR1 mutations are significantly worse compared to those with other forms of endocrine resistance [7, 8, 13].

Following the development of endocrine resistance, patients with MBC are treated with chemotherapy. Paclitaxel and doxorubicin are being among the most commonly used drugs [14]. Yet, some patients rapidly develop resistance to them. Mechanisms associated with resistance to chemotherapy include the activation of survival pathways, such as PI3K/AKT/mTOR, and alterations in drug metabolism, including drug uptake, efflux, and detoxification [15].

Multiple drug resistance (MDR) refers to the ability of cancer cells to resist a variety of chemotherapeutic drugs. The MDR phenotype occurs most often due to the overexpression of drug-efflux pumps in the plasma membrane of cancer cells [16], including the multidrug resistance-1 (*MDR1*) gene, which is known as *ABCB1*. It encodes for P-glycoprotein (P-gp), which is an efflux transporter that limits drugs from penetrating cells and depositing them into the extracellular space [17]. Expression of *MDR1* was noted in over 50% of cancers with MDR phenotype and it can be inherited or induced by chemotherapy [18]. A well-established regulator of the MDR phenotype is c-Jun NH_2_-terminal kinase (JNK) pathway. Exposure of tumors to chemotherapy activates JNK [19], leading to phosphorylation and activation of its downstream effector, the transcription factor c-Jun [20], which in turn upregulates the expression of *MDR1* [21, 22].

Based on the accumulating clinical data, we hypothesized that *ESR1* activating mutations confer resistance to chemotherapy. Indeed, our results indicate an association between these mutations and resistance to chemotherapy in BC cells. Furthermore, *MDR1* was upregulated in mut-ER, especially in D538G-ER, and this was associated with increased activity of the JNK/c-Jun pathway. Importantly, inhibition of this pathway increased sensitivity to doxorubicin. These data suggest a novel treatment option for patients harboring activating *ESR1* mutations.

## Materials and methods

### Chemicals and reagents

Paclitaxel (Taxol; 6 mg/ml) and doxorubicin (2 mg/ml) were provided from the pharmacy of the Oncology department at Sourasky Medical Center (Tel Aviv, Israel). SP600125 (10 mg) ≥ 98% HPLC and JNK inhibitor X (BI-78D3; 5 mg) were purchased from Sigma-Aldrich (St. Louis, MO, USA).

### Cells

Cell lines were originally obtained from the American Type Culture Collection (ATCC) and authenticated with the DNA markers used by ATCC. MCF-7 (RRID: CVCL 0031) and T47D (RRID: CVCL 0553) cells were grown in Dulbecco's Modified Eagle's Medium (DMEM) containing 10% fetal bovine serum (FBS). MCF-7 cell lines stably expressing WT-ER, D538G-ER, and Y537S-ER, were generated in the lab using lentiviral infection [2, 12].

### Methylene blue assay

Viability and proliferation were assessed using methylene blue assay as previously described [12]. For this assay, MCF-7 and T47D cells (WT-ER, D538G, or Y537S expressing cells) were plated in 96 well plates at a density of 5000 cells per well and treated with various concentrations of chemotherapy drugs (10 wells per treatment) for 72 h. To end the assay, glutaraldehyde (2.5%) was diluted 1:5 into cells for 10 min, washed thoroughly three times with ddH_2_O, and then incubated with 100 μl of methylene blue stain [1% methylene blue in borate buffer (pH 8.5)] for 1 h at room temperature. After removing the methylene blue stain, cells were washed with dH_2_O to completely remove the stain and 100 μl of 0.1 M HCl was added into each well following dryness. Subsequently, the absorbance was read with a microplate reader at 650 nm.

### Colony formation assay

MCF-7 WT-ER and mut-ER cells were cultured at low density (1500 cells/well) in 6-well plates and treated twice a week with different concentrations of chemotherapies (0.5 nM paclitaxel, 5 nM doxorubicin) for two weeks. To end the assay, cells were fixed and stained with 0.01% crystal violet diluted with 95% ethanol for 40 min. Quantification of colonies was done by dissolving in 10% acetic acid and read in a plate reader at 560 nm wavelength.

### Luciferase assay

MCF-7 cells were plated in 12-well plates (70,000 cells/per well) and transfected with the AP-1 reporter vector (3XAP1PGL3). Luciferase assay was conducted using the Luciferase Assay System kit (Promega, CA) according to the manufacturer's instructions. Briefly, 100 μL of Luciferase Assay Reagent was injected to each well, and luminescence was immediately measured using a microplate reader. Luciferase units were normalized to total protein concentration determined by Bradford assay.

### Apoptosis assay

To assess the effect of chemotherapy treatment on cell cycle and apoptosis, MCF-7 cells stably expressing WT-ER and the mutations were evaluated by measuring 7AAD and Annexin-FITC V staining using fluorescence-activated cell sorter (FACS Caliber Becton Dickinson). Apoptosis was determined by flow cytometry analysis using annexin-V FITC in accordance with the manufacturer’s instructions (Invitrogen). Briefly, MCF-7 (WT and mut-ER) cells were treated with 20 nM of paclitaxel or regular DMEM media (for control samples). After 72 h of treatment, adherent cells were harvested, washed three times with PBS, and then resuspended in 200 μL binding buffer containing 5 μL of annexin-V fluorescein isothiocyanate and 5 μL of 7AAD, then incubated for 15 min in the dark at room temperature. Analysis was immediately performed using flow cytometer. Data was analyzed by FlowJo software.

### Quantitative RT-PCR

Genes expression was evaluated as previously stated [12]. Briefly, the total RNA was extracted using the High Pure RNA Isolation Kit (Roche). Total RNA (1 μg) was reverse transcribed using qScript cDNA synthesis kit (Quanta Biosciences). Quantitative RT-PCR (qRT-PCR) was used to determine mRNA level. Primers were synthesized by IDT (Coralville, IA, USA). Amplification reactions were performed with Platinum qPCR SuperMix in triplicate using StepOne Plus (Applied Biosystems). PCR conditions: 50 °C for 2 min, 95 °C for 2 min, followed by 40 cycles of 95 °C for 15 s, 60 °C for 45 s. The primer sequences for the genes were as follows: *MDR1*: F-TTCAACTATCCCACCCGACCGGAC, R-ATGCTGCAGTCAAACAGGATGGGC; c-Jun: F-CAGCCAGGTCGGCAGTATAG, R-GGGACTCTGCCACTTGTCTC; β-actin: F-GCTCAGGAGGAGCAATGATCTT; R-TTGCCGACAGGATGCAGAA.

### Western blot

Cells were harvested, lysed, and the total protein was extracted with radioimmunoprecipitation assay (RIPA) buffer (50 mM Tris–HCl pH 7.4, 150 mM NaCl, 1% NP-40,0.25% Na-deoxycholate, 1 mM EDTA, 1 mM NaF), together with a protease and phosphatase inhibitor cocktails (Sigma). Lysates were resolved on 10% SDS-PAGE and immunoblotted with the indicated antibodies:

β-actin (A5441; sigma, St. Louis, MO); p-JNK (AF1205; R&D systems, Minneapolis, United States), p–c-Jun (ser73) (cat# 3270;cell signaling, Massachusetts, United States), T-JNK (cat# 9252;cell signaling, Massachusetts, United States), anti *P*-glycoprotein (ab170904; Abcam, Cambridge, United Kingdom), T-ERK (M-5670; Sigma, St. Louis, MO), c-PARP (cat# 5625; cell signaling, Massachusetts, United States).

### Chromatin immunoprecipitation (ChIP) assay

The assay was performed using Magna ChIP A/G Kit (EMD Millipore Corporation). Cells were grown in 150 mm plates and cross-linked with 1% formaldehyde. Following sonication, chromatin was immunoprecipitated overnight with c-Jun antibody (60A8; Cell Signaling). Normal rabbit IgG (Jackson ImmunoResearch Laboratories, INC) was used as a control. Total DNA was extracted and samples were analyzed on 1.5% agarose after 30 cycles of PCR amplification with primers spanning the AP-1 site on *MDR1* promoter: (F) 5’ CCTCCTGGAAATTCAACCTTG-3’, (R) 5’-GAAGAGCCGCTAGAATG-3’ as previously studied [24].

### Immunohistochemistry (IHC)

The slides were deparaffinized in xylene (Bio-Lab Itd) and rehydrated in graded concentrations of alcohol. Antigen retrieval was performed using a 10 mM sodium citrate buffer solution at pH 6.0. Sections were placed in a 3% hydrogen peroxide for 30 min to quench any endogenous peroxidase activity, followed by several washes of PBS with Tween 20 (PBST) and then incubated with normal horse serum 2.5% (ImmPRESS universal polymer kit peroxidase) for 20 min. Then, the slides were incubated with primary antibody which was diluted with an antibody diluent (Zytomed Systems) overnight. Antibodies used: anti p-JNK (R&D cat# AF1205; 1:100) and P-gp (abcam cat#ab170904; 1:100). Next, slides were incubated with horseradish peroxidase (HPR) for 30 min followed by two washes of PBS with Tween 20 (PBST) and stained with diaminobenzidine (DAB) (ImmPACT DAB Kit) and hematoxylin (Merck). For quantification, number of p-JNK and P-gp cells per field, 4 fields per group, were determined using ImageJ. The P-gp score in patients' clinical samples was interpreted on the basis of percentage of positive cells against total population of cells as previously described [24].

### Intracellular accumulation of doxorubicin

**Confocal microscopic observation.** MCF-7 cells stably expressing WT-ER and D538G were seeded at a density of 50,000 cells onto the coverslips of 12-well plates. After 24 h, cells were treated with 10 μM doxorubicin alone or in combination with a JNK inhibitor, SP600125, (20 μM) for 3 h. Cells were fixed with 4% paraformaldehyde in PBS for 10 min, followed by three washes with PBS. Then cells were permeabilized with 0.1% Triton X-100 in PBS for 5 min at room temperature and followed by three washes of PBS, 10 min each. After blocking with Casein Block (CAS-Block from Invitrogen), cells were stained with anti- EpCAM antibody (abcam, 1:500 diluted with CAS-Block) overnight at 4 °C. After three washes with PBS, the sections were incubated with goat anti-rabbit AlexaFluor 488 antibody (Jackson ImmunoResearch Laboratories, 1:200 dilution) for 1 h at room temperature. Following three washes with PBS, 4,6-diamidino-2-phenylindole (DAPI) (Rhenium) was added for 2 min, followed by two washes with PBS. The coverslips were wet mounted using Fluoromount aqueous mounting (Sigma) on microscope slides. Slides were observed under LSM 700 confocal laser scanning microscope (Zeiss, Germany) using the 40x magnification.

**Flow cytometry**. To quantify the intracellular accumulation of doxorubicin, WT-ER and D538G cells were seeded in 6 well plates. After 24 h, cells were treated with 10 μM doxorubicin alone or in combination with SP600125 (20 μM) for 24 h. The control samples were incubated without any treatment. Then, samples were washed twice with PBS, harvested, and the fluorescence intensity was determined using flow cytometry (FACScantoII; Becton Dickinson, NJ, USA). A minimum of 10,000 events were collected for each sample. The data were analyzed with FlowJo software. The fluorescence intensity was expressed as Geometric Mean.

### Patients clinical data and tumor specimens

Tumor samples were provided in the form of formalin-fixed paraffin-embedded (FFPE) blocks after a written informed consent was obtained from the research subjects by the Tel Aviv Sourasky Medical Center, under an approved institutional review board (IRB) (0137-21-TLV). Clinical data was obtained from patients' electronic medical records.

### Mice tumor xenograft study

Mice maintenance and experiments were carried out under institutional guidelines of the Sourasky Medical Center in accordance with current regulations and standards of the institution Animal Care and Use Committee. We used a subcutaneous mouse model to test the tumorigenic properties and chemoresistance of WT-ER, and D538G-ER-stably expressing cells. Six-week old female athymic nude mice were purchased from Envigo RMS (Jerusalem, Israel). The mice were housed and maintained in laminar flow cabinets under specific pathogen-free conditions. One week prior to cell inoculation, estrogen pellets (0.36 mg/pellet, 90-days release; Innovative Research of America, Sarasota, Florida, USA; Cat. No. SE-121) were implanted subcutaneously on the dorsal side below the shoulder blades. Tumors were induced by injecting 5 × 10^6^ cells/200 μL PBS with Matrigel (1:1 ratio) to mouse flank, 5–6 mice per group. When tumor size reached 100–150 mm^3^, mice were treated with paclitaxel (Taxol; obtained from the Institution pharmacy) or control PBS intraperitoneally. Paclitaxel was given at a dose of 20 mg/kg/week, once a week, for six consecutive weeks. Tumors were measured twice weekly using a caliper by the ellipsoid volume calculation formula 0.5 × (length × width^2^).

### UCSC cancer genomics browser analysis

The heat map and correlation between *ABCB1* (*MDR1*) and c-Jun were constructed by data mining in the Tumor Cancer Genome Atlas (TCGA) breast cancer using the UCSC Xena browser (http://xena.ucsc.edu/).

### Statistical analysis

Statistical analysis was performed using the GraphPad Prism software (GraphPad Software): One Way or Two-Way ANOVA with multiple comparisons Bonferroni post hoc analysis and considered significant at *P*-values * ≤ 0.05, ** ≤ 0.01 and *** ≤ 0.001 Bar graphs represent mean and standard deviation (SD) across multiple independent experimental repeats.

## Results

### LBD mutations are associated with decreased sensitivity to chemotherapy

**Fig. 1:**
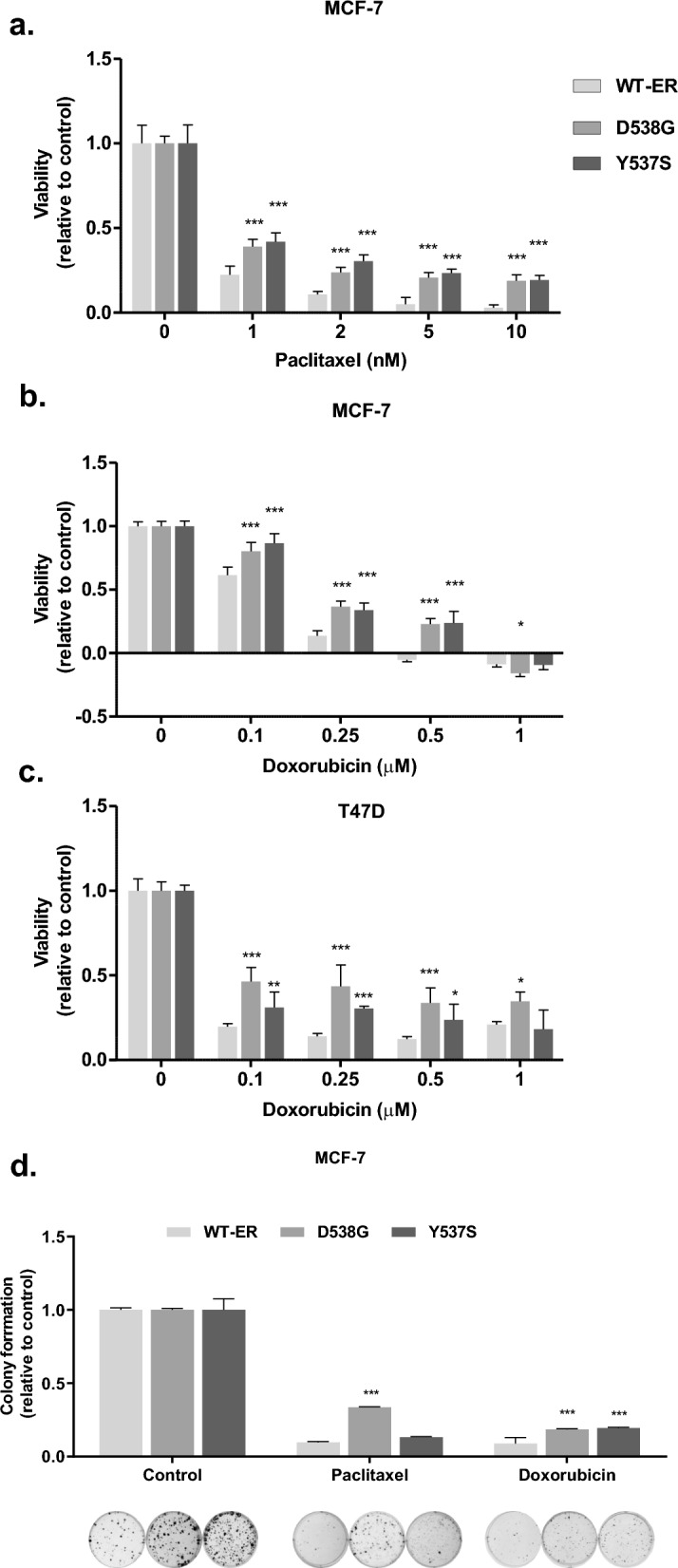
LBD mutations associated with decreased sensitivity to chemotherapy. **a**–**b** MCF-7 WT-ER and LBD-ER cells were seeded in 96 well plates and treated with indicated concentrations of paclitaxel and doxorubicin for 72 h. Viability was assessed using methylene blue assay. **c** T47D WT-ER and LBD-ER cells were seeded in 96 well plates and treated with indicated concentrations of doxorubicin for 72 h. Viability was assessed using methylene blue assay. **d** MCF-7 WT-ER and LBD-ER cells were seeded at low density and then treated with chemotherapy drugs twice a week for 2 weeks, then cells were fixed and colonies stained with crystal violet. Quantification of colonies was done by dissolving in 10% acetic acid and read in a plate reader at 560 nm wavelength. *P < 0.05, **P < 0.01, ***P < 0.001. Each bar represents the mean ± SD. Experiment was repeated 3 times, and a representative experiment is depicted

We first aimed to study the sensitivity of BC cells harboring ESR1 activating mutations to doxorubicin and paclitaxel, some of the most commonly used drugs in MBC [25]. Cells expressing mutated ER (mut-ER) showed increased resistance to the drugs (Fig. 1a–b). Thus, paclitaxel at 1 nM decreased viability of MCF-7 WT-ER and mut-ER cells by 80 and 50%, respectively (Fig. 1a,* p *< 0.001), and significant differences were observed for all paclitaxel doses. Similarly, none of the WT-ER cells survived 0.5 μM doxorubicin, compared to ~ 30% of mutated cells (Fig. 1b,* p* < 0.001). Furthermore, similar results were observed in T47D cells expressing WT-ER, D538G, and Y537S cells. Significant differences were observed for all doxorubicin doses in the mutated cells compared to WT-ER cells (Fig. 1c). Moreover, colony formation assay using 0.5 nM paclitaxel and 5 nM of doxorubicin (Fig. 1d) yielded similar results. Indeed, mutated cells formed more colonies than WT-ER cells, and the relative decrease in colony number and size was significantly greater in WT-ER cells compared to the mutated cells. Paclitaxel treatment increased colony formation three-folds in the D538G mutated cells compare to WT-ER and Y537S cells (Fig. 1d, * p* < 0.001). While doxorubicin treatment reduces colony numbers by 90% in WT-ER compared to 70% in both D538G and Y537S cells (Fig. 1d, * p *< 0.001).

### LBD-ER mutant cells are resistant to chemotherapy-induced apoptosis

**Fig. 2:**
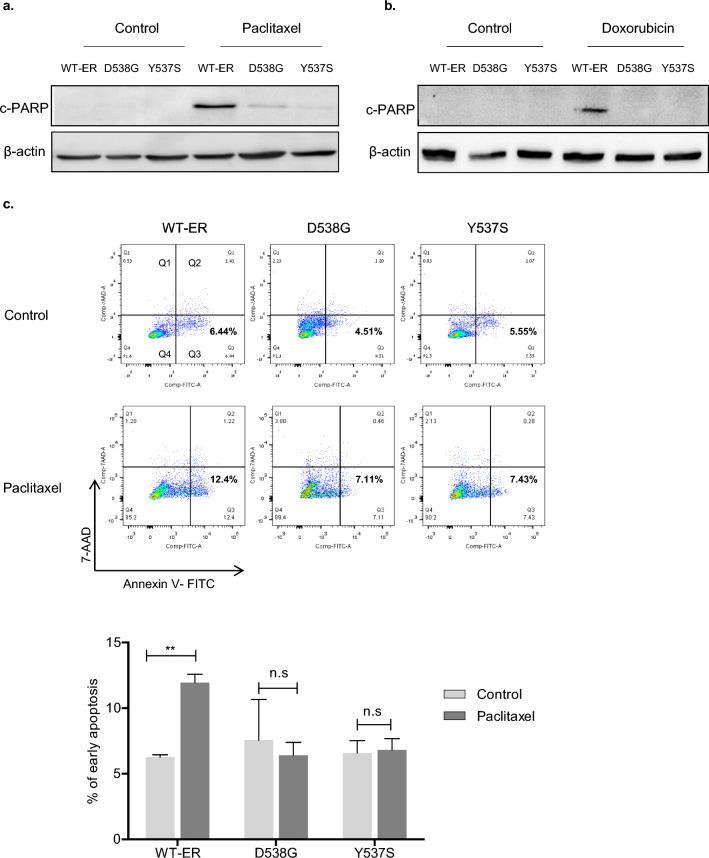
LBD-ER mutant cells are resistant to chemotherapy-induced apoptosis. **a**–**b** MCF-7 WT-ER and LBD-ER cells were treated with 20 nM paclitaxel or 200 nM doxorubicin for 72 h. Cells were lysed and immunoblotted with c-PARP. β-actin served as a control. **c** MCF-7 cells were treated with 20 nM paclitaxel for 72 h then stained with Annexin/7AAD according to the manufacturer's protocol and analyzed by flow cytometry. Bar chart demonstrated the percentage of early apoptosis in cells as. Data represent the mean value of three independent experiments. Each bar represents the mean ± S.D. **P < 0.01

As part of the characterization of chemoresistant cells, we examined the role of apoptosis in MCF-7 cells expressing WT-ER, D538G and Y537S-ER. For this aim, cells were treated with chemotherapy for 72 h and cleaved PARP (c-PARP) levels were monitored. Treatments with paclitaxel and doxorubicin exerted the highest effect on PARP cleavage in WT-ER cells compared to the mut-ER cells (Fig. 2a–b). This observation suggests that mut-ER cells exhibit increased resistance to these chemotherapeutic agents, as evidenced by their diminished apoptotic response relative to WT-ER cells. In addition, apoptosis studies using Annexin V and 7AAD staining indicated that paclitaxel treatment induced early apoptosis of 12.4% in WT-ER cells compared to 7.1% in D538G and 7.4% in Y537S (Fig. 2c). This differential response suggests that the ER mutations may confer some resistance to paclitaxel-induced apoptosis.

### Mutated ER cells are more resistant to paclitaxel* in vivo*

**Fig. 3:**
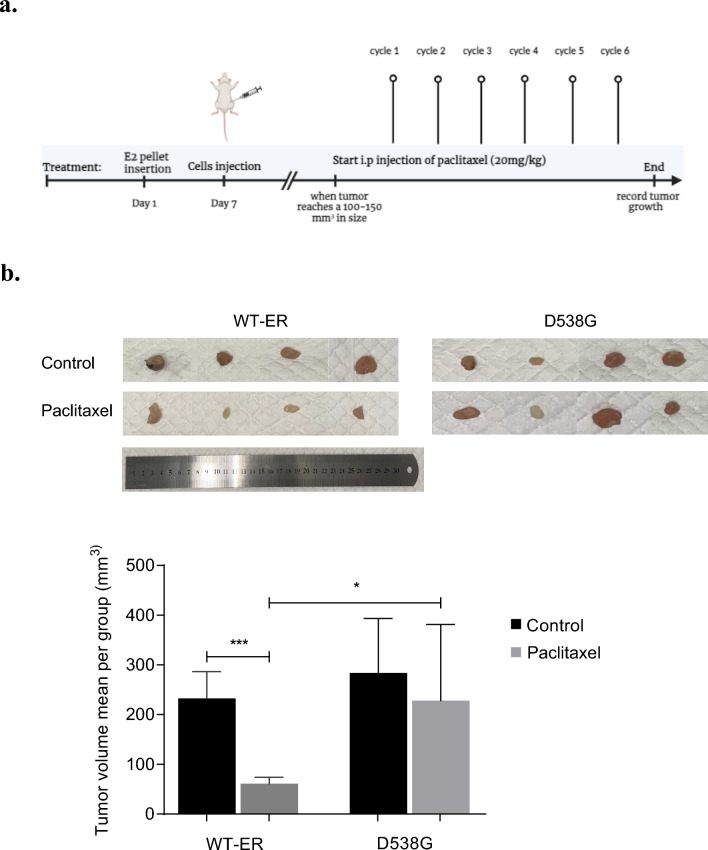
Mutated ER cells are more resistant to paclitaxel in vivo. **a** Experimental design of paclitaxel treatment in vivo. **b** Nude mice were inoculated subcutaneously with WT (*n* = 11) and D538G-ER cells (*n* = 10). When tumor reached 100–150 mm^3^ in volume, mice were divided randomly into groups and paclitaxel was administered for 6 weeks. Tumor size was measured twice weekly. For control groups (*n* = 5 for WT and D538G) and for paclitaxel (*n* = 6 for WT and *n* = 5 for D538G). Each bar represents the mean ± S.D. *P < 0.05; ***P < 0.001

In order to study resistance to chemotherapy in vivo, female nude mice were first supplemented with estrogen pellet and then injected with D538G or WT-ER MCF-7 cells (Fig. 3a) and treated with either vehicle control or paclitaxel. While paclitaxel significantly inhibited the tumor growth of the WT-ER group (Fig. 3b,* p *< 0.0001), resistance to paclitaxel was noted in the D538G mice group, and they formed larger tumors compared to WT-ER (Fig. 3b,* p* < 0.05).

### Upregulation of the MDR1 in the D538G-ER mutant cells

**Fig. 4:**
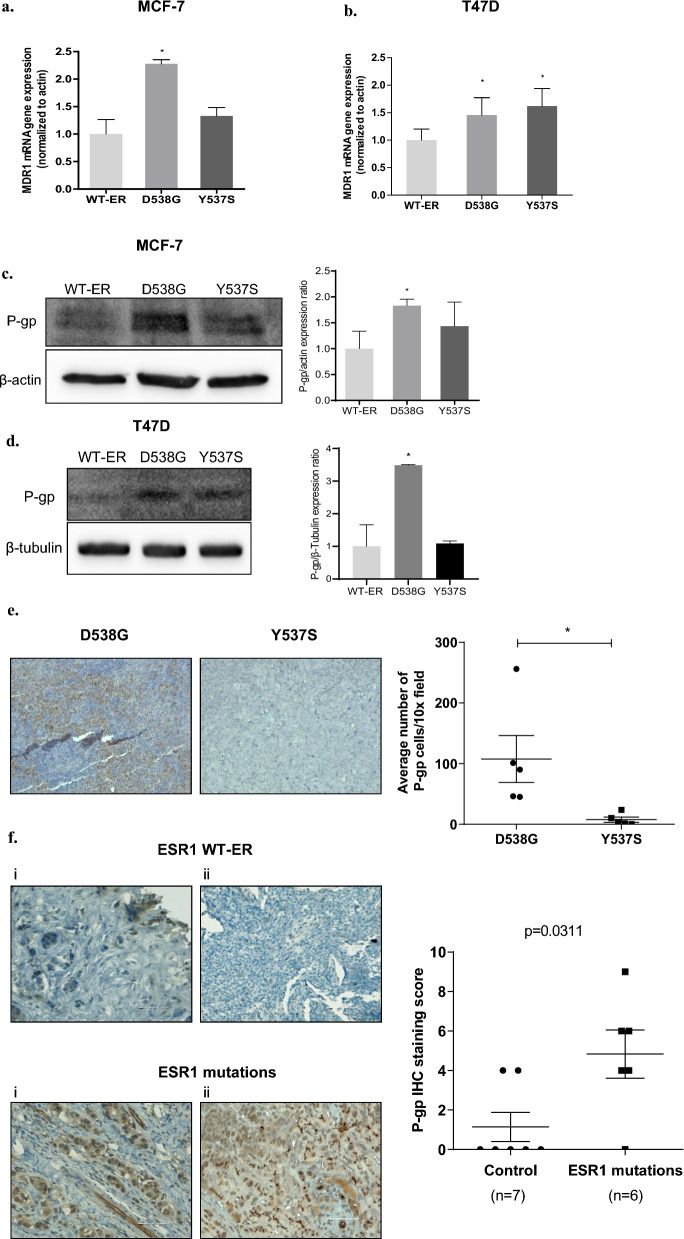
Upregulation of the MDR1 in the D538G-ER mutant cells. **a** MDR1 mRNA expression level in MCF-7 WT-ER and the LBD-ER cells was determined by q-RT-PCR. **b** Expression of MDR1 mRNA in T47D cells expressing WT-ER, D538G and Y537S cells determined by qRT-PCR. Values were normalized to β-actin. P-gp protein level was evaluated and quantified in MCF-7 cells (**c**) and T47D cells (**d**) using western blot. β-actin was used as a loading control.** e** IHC staining of P-gp expression in mice tumor samples, D538G (*n* = 5) and Y537S (*n* = 5). Representative photomicrographs (taken with a 10 × objective). P-gp relative levels were determined by taking the average of 4 fields per group using Image J. **f** Representative IHC staining of P-gp protein levels in BC metastasis with ESR1 mutations (*n* = 6) and WT-ESR1 (*n* = 7). A dot-plot of IHC data quantification (mean ± s.d., unpaired *t*-test). Scale bars represent 80 µm. *P < 0.05, **P < 0.01

Overexpression of *MDR1* commonly mediates resistance of cancer cells to paclitaxel and doxorubicin [26]. Therefore, we aimed to study the expression of *MDR1* mRNA and the protein it encodes, P-gp, in MCF-7 and T47D WT-ER and mutant cells. *MDR1* mRNA expression level was 2.3-fold higher in D538G-ER cells (*p* < 0.05, Fig. 4a) and 1.3 in Y537S-ER MCF-7 cells.

Of note, *MDR1* expression significantly increased following doxorubicin treatment in the D538G cells (*p* < 0.05, supplementary Fig. S1). We also observed increased levels of the encoded protein P-gp were noted in the D538G-ER cells compared to both Y537S and WT-ER cells (Fig. 4c). In order to validate this differential effect of the two mutations and eliminate the possibility of clonal effect, expression of the *MDR1* gene was also studied in T47D cells expressing either WT-ER, D538G, or Y537S and revealed a similar pattern of *MDR1* mRNA and P-gp protein expression (Fig. 4b and d).

Moreover, to corroborate these results, we assessed P-gp levels using IHC in tumor samples derived from an orthotopic mouse model injected with D538G or Y537S ER cells (generated by us previously and described in detail [12]) and noted higher P-gp expression in D538G-ER tumor cells compared to Y537S-ER (Fig. 4e). Tumors formed by WT-ER cells were very small and could not be used to assess the P-gp expression.

Next, we aimed to assess the expression profile of P-gp across MBC clinical samples with and without ESR1 mutations. We obtained clinical samples of metastatic breast cancer tumors derived from patients with endocrine resistance, either harboring the ESR1 mutations (*n* = 6), or with WT-ER (*n* = 7). Our results showed a significant elevation in P-gp expression within ESR1 mutation group compared to the control (*p* = 0.0311, Fig. 4f and Supplementary Fig. S2). These patients mostly harbor the D538G mutations that metastasize to either bone or liver (Supplementary Table 1). Our results showed increased expression of P-gp was associated with liver metastasis. To strengthen the association between ESR1 mutations and liver metastasis, we analyzed 1918 tumor samples from a publicly available primary and metastatic breast cancer dataset. We focused on 495 metastatic samples that were obtained from bone, liver, lung, or lymph nodes. We conclude that while ESR1-WT tumors disseminate in similar proportion between the different sites, ESR1 mutation changes the pattern of metastasis (Supplementary Table 2, *P*-value = 3.804366 × 10–05) with liver being 2–3 times more prevalent than the other metastatic sites.

### JNK/c-Jun signaling pathway is elevated in the mut-ER cells

**Fig. 5:**
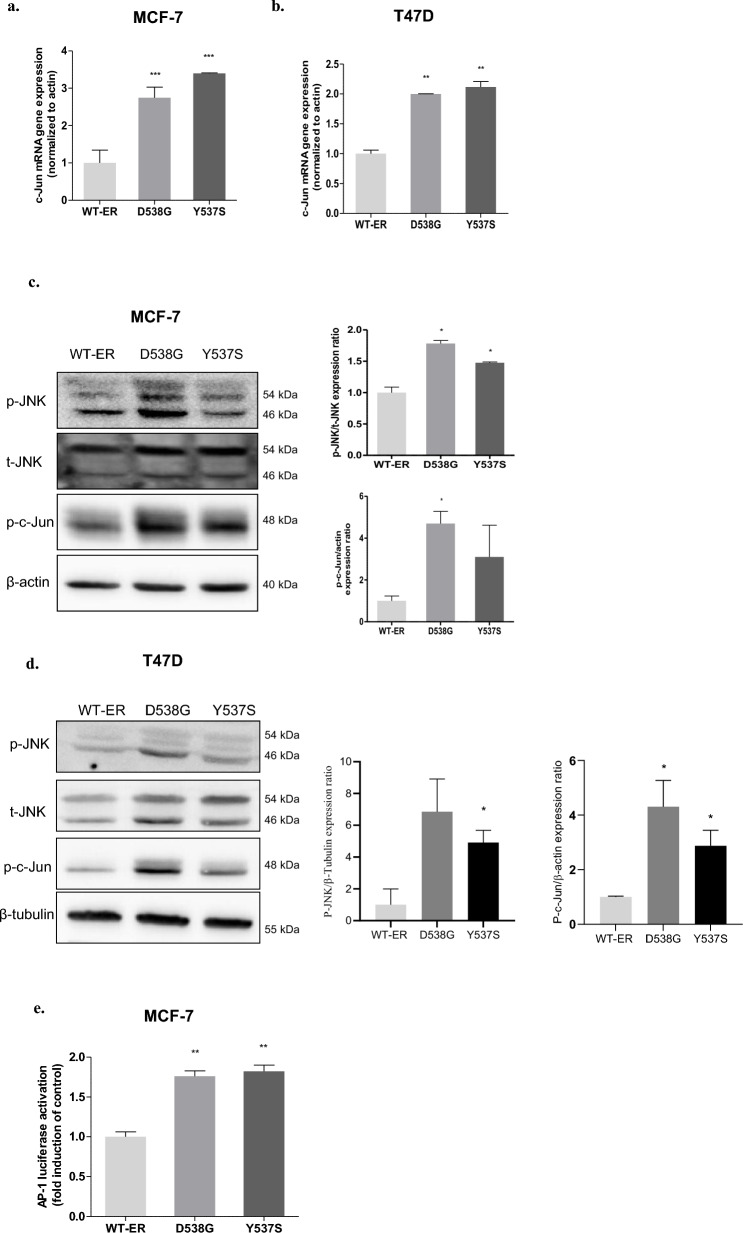

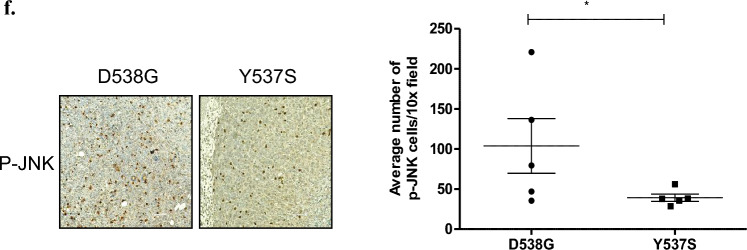
JNK/c-Jun signaling pathway is elevated in the mut-ER cells. **a** c-Jun mRNA expression level in MCF-7 WT-ER and the LBD-ER cells was determined by q-RT-PCR. Values were normalized to β-actin. **b** Expression of c-Jun mRNA in T47D cells expressing WT-ER, D538G and Y537S cells determined by qRT-PCR. **c** p-JNK (T183/Y185), T-JNK, and p–c-Jun (ser73) protein levels were evaluated using western blot. β-actin was used as a loading control. Quantification of the p-JNK and p–c-Jun levels are shown in the histograms. **d** Expression of p-JNK, p–c-Jun, and t-JNK proteins in T47D cells was determined by western blot and quantification of the p-JNK and p–c-Jun levels are shown in the histograms. **e** MCF-7 WT-ER and the LBD-ER cells were transfected with AP-1-luciferase reporter plasmid. After 24 h, luciferase assay was performed, and results were normalized to protein concentration. **f** IHC analysis of p-JNK expression in mice tumor samples (D538G (*n* = 5) and Y537S (*n* = 5)) using DAB staining. Representative photomicrographs (taken with a 10 × objective). p-JNK relative levels were quantified by taking the average of 4 fields per group using Image J. Scale bars represent 80 µm. Each bar represents ± SD, statistical analysis was performed using unpaired *t*-test (*P < 0.05, **P < 0.01, ***P < 0.001 compared to WT-ER control)

*MDR1* expression is known to be regulated by the JNK/c-Jun signaling pathway [21, 27, 28] and this pathway is implicated in conferring chemoresistance [29–31]. First, we studied c-Jun mRNA levels in MCF-7 cells and found 2.8 and 3.4-fold higher expression in D538G and Y537S-ER cells, respectively (Fig. 5a). Furthermore, a similar trend was observed in T47D cells (Fig. 5b) Next, we examined the expression of phosphorylated (p)-JNK and c-Jun in mut-ER cells. We found that mut-ER cells, especially D538G cells, expressed higher levels of p-JNK and p–c-Jun compared to WT-ER cells in both MCF-7 and T47D cells (Fig. 5c–d). In order to eliminate the possibility of clonal effects, we studied the expression of p-JNK and p–c-Jun in additional MCF-7 mut-ER clones and observed a similar higher expression of these proteins in these clones (Supplementary Fig. S3). To further investigate the impact of the mut-ER cells on cellular signaling pathways, we aimed to assess c-Jun transcriptional activity key component, AP-1 transcription factor complex. This complex plays a crucial role in regulating various cellular processes, including proliferation, differentiation, and apoptosis. Therefore, to quantify the differences in c-Jun activity between MCF-7 WT-ER and mut-ER cells, we employed an AP1-luciferase reporter gene assay. Our results revealed a nearly two-fold increase in AP-1 transcriptional activity in the MCF-7 mut-ER cells compared to WT-ER cells (*p* < 0.01, Fig. 5e). In accordance with these results, IHC staining of mice tumor samples that were injected with the mut–ER cells, as previously described [12], showed higher p-JNK expression in D538G tumors compared to Y537S tumors (Fig. 5f).

### JNK/c-Jun pathway upregulates MDR1 expression, especially in D538G mutant cells

**Fig. 6:**
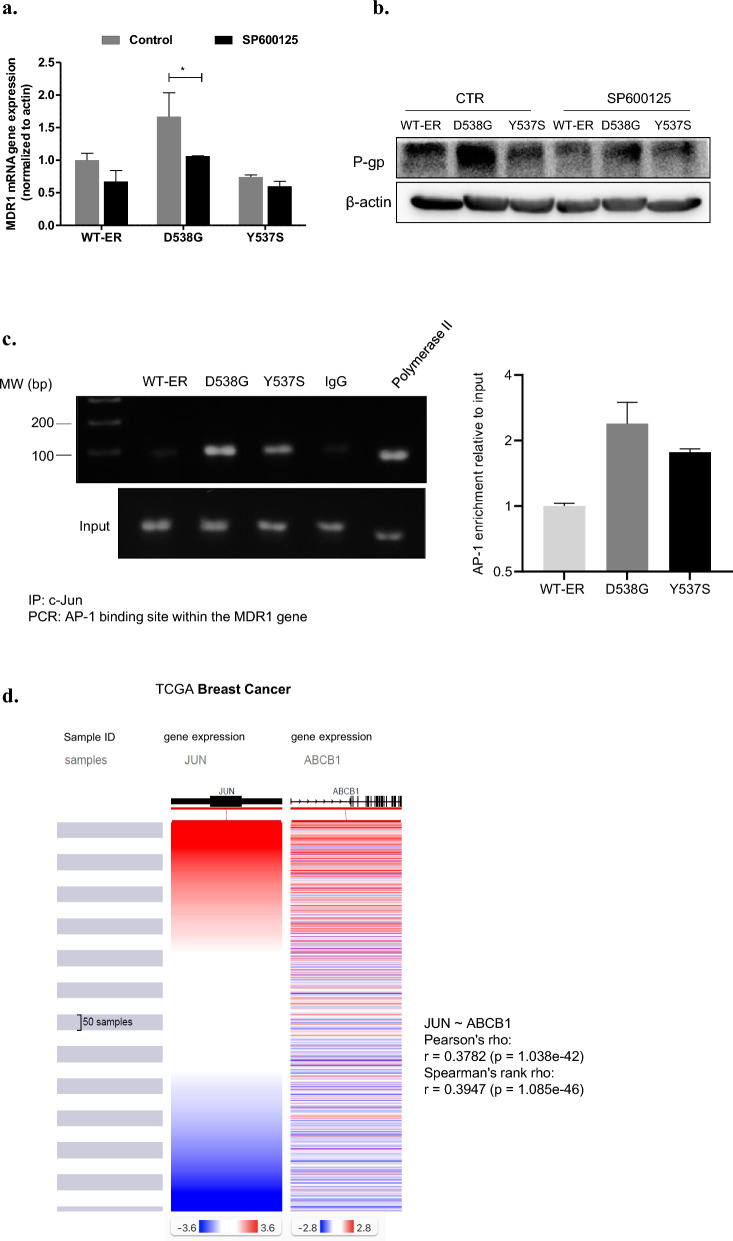
JNK/c-Jun pathway regulates MDR1 expression in mut-ER cells. **a** MCF-7 WT-ER and LBD-ER cells were treated with 20 μM of SP600125 (SP) for 24 h and the MDR1 mRNA expression level was determined by q-RT-PCR. Values were normalized to β-actin. **b** Expression of P-gp was determined by western blot after treatment with 20 μM of SP600125 (SP) for 24 h. **c** ChIP analysis with anti c-Jun antibody for immunoprecipitation was carried out with primers for the AP-1 site on the MDR1 promoter. Normal rabbit IgG was used as a negative control and polymerase II was used as a positive control. Quantification of the AP-1 enrichment relative to Input is shown in the histogram. **d** Correlation analysis between ABCB1 and JUN gene expressions in breast cancer was conducted using BRCA TCGA dataset, using Xena browser. Correlation analysis was performed using Pearson's and Spearman's correlation. (Red represents high expression of gene, blue represents low expression of gene). *P < 0.05. Each bar represents the mean ± SD. Experiment was repeated 3 times and a representative experiment is depicted

As studies have shown that the JNK/c-Jun signaling pathway induces *MDR1* expression [21, 27, 28], we hypothesized that JNK inhibition would reduce *MDR1* expression. To confirm this effect, we inhibited JNK using a commonly used inhibitor, SP600125 (SP). Our results show that its inhibition decreased *MDR1* mRNA (Fig. 6a) and protein levels, especially in D538G cells (Fig. 6b). Similar results were produced when treated with an additional JNK inhibitor, BI-78D3 (Supplementary Fig. S4a–b). We confirmed the potency of these inhibitors by monitoring p–c-Jun expression (Supplementary Fig. S5). These results suggest that JNK activity plays a role in regulating *MDR1* expression. Particularly, D538G cells appear to be more sensitive to JNK inhibition, which was consistent across different inhibitors (SP600125 and BI-78D3).

These results have important implications for understanding and potentially targeting multidrug resistance in cancer cells, as *MDR1* is known to contribute to drug resistance mechanisms.

Therefore, our next aim was to determine if the JNK/c-Jun pathway plays a direct role in regulating *MDR1* expression. To achieve this, we employed a Chromatin Immunoprecipitation (ChIP) assay, which allowed us to examine whether c-Jun directly interacts with and binds to the promoter region of the *MDR1* gene. We performed a PCR, flanking the AP-1 binding site in the *MDR1* promoter region, following c-Jun immunoprecipitation. Results showed a PCR product in D538G and Y537S–ER mutant cells, yet AP-1 enrichment was higher in D538G-ER cells, and no enrichment was observed in the WT-ER cells (Fig. 6c). These results suggest that c-Jun directly transcribes the *MDR1* gene in mut-ER cells, mainly in the D538G mutated cells. Collectively, our results showed a more pronounced increase in JNK/c-Jun and *MDR1* expressions in D538G mutations in both MCF-7 and T47D cells. These data emphasize that the two mutations differ in their mechanisms of chemoresistance. Importantly, we verified that ER does not directly induce *MDR1* expression. We analyzed 3000 bp upstream of the *MDR1* promoter and did not detect classical ER binding sites (conducted using http://gene-regulation.com/pub/programs/alibaba2/).

In order to study the relationship between *ABCB1* (*MDR1*) and *JUN* expressions in breast cancer clinical samples, we analyzed TCGA transcriptomic databases. Using the UCSC Xena Browser (http://xena.ucsc.edu/), we found a positive correlation between *JUN* and *ABCB1* (*r* = 0.3782, *p* = 1.038e − 42 based on Pearson's correlation and *r* = 0.3782, *p* = 1.038e − 42 based on Spearman’s correlation) (Fig. 6d).

### JNK inhibition sensitizes breast cancer cells to chemotherapy

**Fig. 7:**
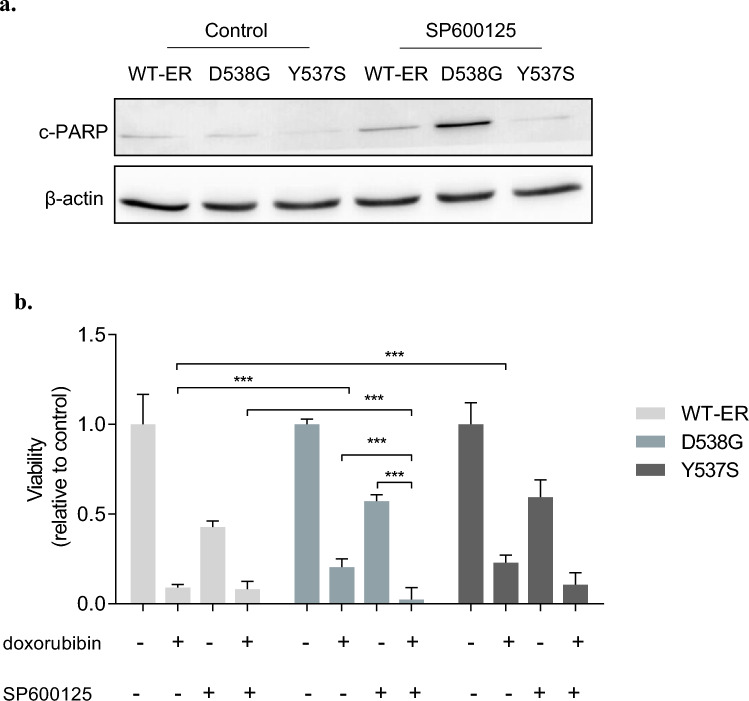
JNK inhibition restores sensitivity of mut-ER and WT-ER cells. **a** MCF-7 expressing the WT-ER and LBD-ER cells were treated with 20 μM of SP for 24 h, then cells were lysed and immunoblotted against c-PARP and β-actin served as a loading control. **b** Cells were seeded in 96 well plates and treated with 20 μM SP, 0.5 μM doxorubicin, or combination for 72 h. Viability was assessed using methylene blue assay. ***P < 0.001. Each bar represents the mean ± SD. Experiment was repeated 3 times and a representative experiment is depicted

We aimed to reveal whether modulation of the JNK signaling pathway can restore sensitivity to chemotherapy in the mut-ER cells. First, we studied the effect of JNK inhibition on apoptosis in MCF-7 mut-ER and WT-ER cells. We treated cells with the JNK inhibitor SP, examined c-PARP expression, and found that JNK inhibition induced apoptosis, mostly in D538G cells (Fig. 7a). In order to determine the role of JNK in mediating chemoresistance in these cells, we compared cell viability after treatment with doxorubicin, SP, or their combination. As expected, the results showed that mut-ER cells were more resistant to doxorubicin treatment (Fig. 7b; *p* < 0.001 compared to WT-ER). Interestingly, while SP inhibited both WT-ER and mut-ER cells, WT-ER cells were more affected (45 vs 55% viability). Importantly, co-treatment with SP and doxorubicin sensitized mut-ER cells to doxorubicin. Of note, co-treatment of WT-ER cells did not reduce cells viability beyond doxorubicin alone, which may be due to the strong effect of doxorubicin as a single treatment (Fig. 7b; *p* < 0.001 of D538G cells when treated with combination compared to doxorubicin alone). On the other hand, there was no significant decrease following combination of SP and paclitaxel compared to paclitaxel alone for both the WT-ER and the D538G cells (Supplementary Fig. S6). Previously, it was shown that one of SP mechanisms of action is similar to that of paclitaxel, namely, it binds tubulin and increases tubulin polymerization disrupting cell cycle progression [32]. This shared mechanism likely accounts for the lack of enhanced efficacy observed with paclitaxel.

### Doxorubicin accumulation is decreased in D538G mutated cells

**Fig. 8:**
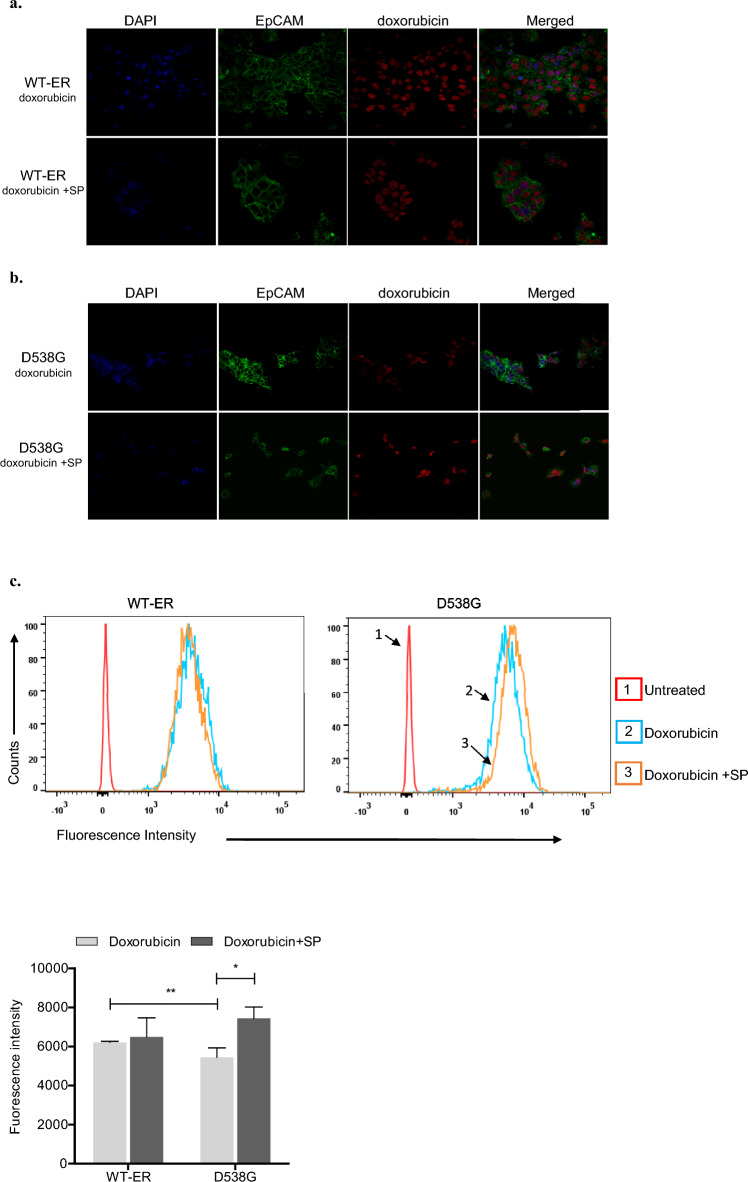

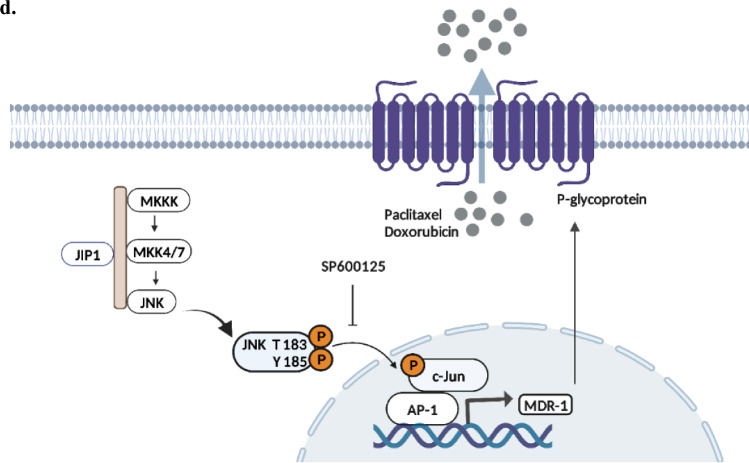
Intracellular accumulation of doxorubicin is decreased in the D538G mutated cells. **a**–**b** Confocal fluorescence microscopy was used to evaluate the intracellular accumulation following the treatment of 10 μM doxorubicin alone and in combination with 20 μM SP for 3 h in MCF-7 WT-ER and D538G cells. Representative images of DAPI, EpCAM, doxorubicin, and merged are shown. Scale bars represent 100 μm. **c** MCF-7 WT-ER and D538G cells were treated with 10 μM doxorubicin alone and with 20 μM SP for 24 h. For flow cytometry measurement, cells were trypsinized, diluted in PBS, and analyzed by FACS. Results are shown as the relative mean fluorescence and represent the average of three independent experiments (mean ± SD). Statistical analyses were conducted using the Student's *t*-test. **e** A schematic representation of the JNK/c-Jun MDR1 signaling pathway in the mut-ER cells. *P < 0.05, **P < 0.01

It has been well established that doxorubicin is one of the P-gp substrates and that [33] P-gp diminishes the internalization and accumulation of doxorubicin in cancer cells, leading to chemoresistance [34]. As we first showed that D538G mutated cells express higher P-gp levels compared to WT-ER, our next aim was to show that doxorubicin accumulation is reduced in D538G mutated cells. To this aim, we monitored cellular accumulation of fluorescently label doxorubicin (DOX-F). We treated MCF-7 WT-ER and D538G cells with DOX-F, and in accordance with our hypothesis, we observed the fluorescent signal in the nuclei of the D538G mutated cells was lower compared to WT-ER cells (Fig. 8a–b). In order to validate that the JNK pathway modulates P-gp expression and hence activity, cells were treated with DOX-F and SP together. Indeed, the intensity of DOX-F fluorescence increased upon the addition of SP in the D538G mutated cells (Fig. 8b), suggesting that the JNK pathway regulates doxorubicin intracellular accumulation.

We studied intracellular accumulation of doxorubicin also using FACS analysis. In agreement with the imaging data, doxorubicin accumulated more in WT-ER cells compared to D538G-ER cells (Fig. 8c,* p* < 0.01). Furthermore, SP treatment increased fluorescence intensity only in D538G cells (Fig. 8c; *p* < 0.05). These results strongly suggest a decrease in doxorubicin accumulation in the D538G cells as they are resistant to doxorubicin than WT-ER cells. 

## Discussion

Approximately 40% of patients with ER-α positive metastatic breast cancer acquire resistance to endocrine therapy due to the acquisition of LBD-ER mutations [35]. Importantly, this group of patients often have a more aggressive disease and worse prognosis [36]. Chemoresistance continues to be a major obstacle that impairs the efficacy of cancer therapy and nearly 90% of patients fail to respond to chemotherapy due to resistance of cancer cells, either de novo or acquired resistance [37]. As most patients with endocrine resistance are treated with chemotherapy, we hypothesized that reduced response to chemotherapy may contribute to the worse prognosis seen in patients harboring these mutations. Yet, no studies have assessed chemotherapy resistance in ER-positive patients harboring LBD-ER mutations. In our study, we discovered that LBD-ER cells are more resistant to chemotherapy drugs, doxorubicin and paclitaxel, compared to the WT-ER cells using both in vitro and in vivo models.

In order to study chemoresistance, we employed the well-established MCF-7 cell model that expresses WT, D538G or Y537S–ER, at physiological levels [12]. Using these cells, we explored the effect of the mutations on viability, colony formation, and apoptosis following chemotherapy treatments. Our results show that LBD-ER cells are more resistant compared to the WT-ER cells. Of note, we established an in vivo model, and observed that paclitaxel treatment led to tumor shrinkage in the WT-ER group only (Fig. 3b). It is important to point out that while the in vivo experiment showed a clear resistance of D538G-ER tumors compared to WT-ER tumors, this model had some limitations. First, we employed subcutaneous implantation approach; thus, drug response may be modulated by different microenvironmental stimuli. Second, we used paclitaxel doses suitable for mice tolerance, and perhaps WT-ER cells would respond to higher doses. Yet, the accumulating data we present strongly suggest a relative resistance to chemotherapy in mut-ER cells.

There are several mechanisms that enable BC cells to acquire drug resistance. Among these are the well characterized P-gp transporters, which are encoded by the *MDR1* gene [38]. Doxorubicin is one of the major substrates of P-gp; therefore, it plays a role in drug internalization within cancer cells [39]. Similarly, low doxorubicin accumulation has been reported in MDR cell lines including MCF-7^ADR^ [40]. Indeed, we have shown that doxorubicin accumulation was lower in the D538G mutated cells compared to WT-ER (Fig. 8b–c). We further revealed that LBD-ER mutant cells exhibited elevated *MDR1* expression, though more prominently in D538G, compared to the WT-ER in both MCF-7 and T47D cells (Fig. 4a–d). In agreement with these results, we found an increase in the P-gp expression level in patients' samples harboring the ESR1 mutations compared to patients without the mutation (Fig. 4f). Our results suggest that the mut-ER cells are able to utilize the P-gp transporters as part of their resistance mechanism to overcome chemotherapy treatments. A relationship between endocrine treatment and P-gp was demonstrated previously, showing that tamoxifen increases sensitivity to doxorubicin, and increases accumulation of vinblastine in MCF-7 cells expressing *MDR1* [41]. On the other hand, another study showed that WW domain-binding protein 2 (WBP2) can bind ER and enhance *MDR1* expression [42].

We aimed to reveal the mechanism through which mut-ER induces *MDR1* expression. Although *MDR1* expression was observed primarily in mut-ER cells, we first confirmed that there are no ER binding sites on the *MDR1* promoter. In search for a possible mechanism, we focused on the JNK/c-Jun pathway as it has been shown to play a role in chemoresistance of some types of cancer [43–45]. ThusIn addition, previous studies revealed that activation of JNKs, leading to increased c-Jun  transcriptional activity , also led to the transcription of other genes related to drug resistance, like XIAP, c-fos, and JunB [46, 47]. Therefore, our goal was to reveal whether the JNK pathway was differentially regulated in LBD-ER cells. We observed activation of the JNK/c-Jun pathway as evidenced by high expressions of p-JNK, p–c-Jun and elevated AP-1 transcriptional activity in the LBD-ER mutant cells compared to WT-ER (Fig. 5). These data suggest a role of the JNK pathway in the LBD-ER mutations. Our results are supported by several studies. Increased JNK signaling was observed following long term acquisition of resistance to endocrine therapies [48]. Another study showed that activation of the JNK pathway is associated with everolimus resistance in endocrine resistant cells [49]. Noteworthy, the important role AP-1 plays in endocrine resistance was demonstrated in a study where inhibition of AP-1, in both in vitro and in vivo settings, enhanced anti proliferative effect of endocrine treatments and delayed the onset of tamoxifen resistance in mice model [50].

The JNK/c-Jun pathway is known to be associated with *MDR1* expression. Several studies have shown that JNK/c-Jun pathway is activated in cancer cells that acquired resistance to chemotherapy in vitro, demonstrating the importance in regulating *MDR1* expression [21, 23, 28]. To test this, we first showed that SP600125, an inhibitor of the JNK signaling pathway, downregulated the expression of P-gp and *MDR1*, especially in the D538G cells. To provide additional evidence, we performed a ChIP assay and indeed our results showed an increase in c-Jun occupancy in the MDR1 promoter, leading to an increase in P-gp transporters in the mutated cells. In addition, a bioinformatics analysis of TCGA breast cancer BRCA database showed a strong positive correlation between *JUN* and *ABCB1*, confirming our results that c-Jun potentiates the *MDR1* expression in BC cells.

The JNK/c-Jun pathway emerged as a differentially activated pathway in mut-ER cells that directly regulates *MDR1* expression. Hence, our next aim was to assess whether this pathway may serve as a novel target for mut-ER breast cancer tumors. We studied viability and apoptosis of WT and mut-ER cells treated with JNK inhibitors and found that JNK inhibition decreased viability in both WT and D538G cells but significantly increased apoptosis in the D538G mutated cells. Previously, it was shown that JNK inhibition decreased cell growth in nasopharyngeal carcinoma [51] and triple-negative breast cancer [52]. As we found that c-Jun directly induces *MDR1* expression, we aimed to reveal whether inhibition of c-Jun activity would restore cell sensitivity to chemotherapy. Indeed, using a JNK inhibitor (SP600125) we were able to sensitize cells to doxorubicin. This effect was observed in all cell lines, with highest effect in D538G-ER cells. Indeed, it was shown that SP600125 reduced viability of breast cancer cells which acquired resistance to doxorubicin [53] and the combined treatment with SP600125 inhibited cell growth more effectively than the single treatments [51]. Indeed, we showed that JNK inhibition led to increase the accumulation of doxorubicin in the D538G-ER cells. These results imply that doxorubicin resistance can be accounted for by decreased amounts of drug at nuclear targets, which may be mediated by the expression and function of P-gp.

Interestingly, while Y537S and D538G exhibit similar behavior, a detailed analysis suggests some subtle, yet potentially important differences. Thus, we observed less JNK phosphorylation in Y537S cells compared to D538G cells (as verified by analyzing different stable clones, supplementary Fig. S2). Additionally, we noted higher *MDR1* expression in D538G cells and lower binding of c-Jun to *MDR1* promoter in Y537S cells (Fig. 6c). These results suggest unique characteristics for each mutation despite their similar role in conferring endocrine resistance. Consistent with our observations, previous studies have also revealed notable differences between Y537S and D538G. For example, Y537S confers higher endocrine resistance than D538G cells and is associated with a shorter overall survival [54].

While we focused on the JNK-MDR1 axis in mut-ER cells, studies showed that resistance to chemotherapy in breast cancer could evolve from a wide array of mechanisms [55], some of them are relevant to mut-ER cells. For example, active PI3K/mTOR pathway may lead to resistance to chemotherapy [56], and several studies including ours [12, 57] showed increased activation of this pathway in mut-ER cells.

## Conclusions

Taken together, our current study demonstrates that activating mutations in the LBD-ER not only confer resistance to endocrine therapy but also relative resistance to commonly used chemotherapy. This resistance is, at least in part, mediated by the activation of the JNK/c-Jun pathway, leading to the upregulation of *MDR1* expression (Fig. 8d). These findings underscore the urgency of discovering new drugs to effectively treat patients with ESR1-mutated tumors, as these tumors display decreased responsiveness even to subsequent lines of treatment such as chemotherapy. Moreover, the study highlights the significance of targeting the JNK/c-Jun as a strategy to sensitize mut-ER cells to chemotherapy. Furthermore, this study reveals distinct differences between the Y537S and D538G mutations, emphasizing the importance of carefully examining these discrepancies, as they may hold implications for the treatment of patients harboring these specific mutations.

## Supplementary Information

Below is the link to the electronic supplementary material.

Supplementary file1 (PPTX 3695 KB)

## Data Availability

The datasets used and/or analyzed during the current study are available from the corresponding author on reasonable request.

## Abbreviations

BC: Breast cancer
MBC: Metastatic breast cancer
ERα: Estrogen receptor-a
LBD: Ligand-binding domain
MDR1: Multiple drug resistance-1
P-gp: P-glycoprotein
JNK: C-Jun N-terminal kinase
