# Supplementary material for: Beyond endocrine resistance: estrogen receptor (ESR1) activating mutations mediate chemotherapy resistance through the JNK/c-Jun MDR1 pathway in breast cancer
Source: Breast Cancer Res Treat. 2024 Oct 29;209(2):431–49. doi: 10.1007/s10549-024-07507-3 (PMC11785692; doi:10.1007/s10549-024-07507-3)
Supplement: Supplementary file 1 — Supplementary file1 (PPTX 3695 KB) [file 10549_2024_7507_MOESM1_ESM.pptx]

## Slide 1
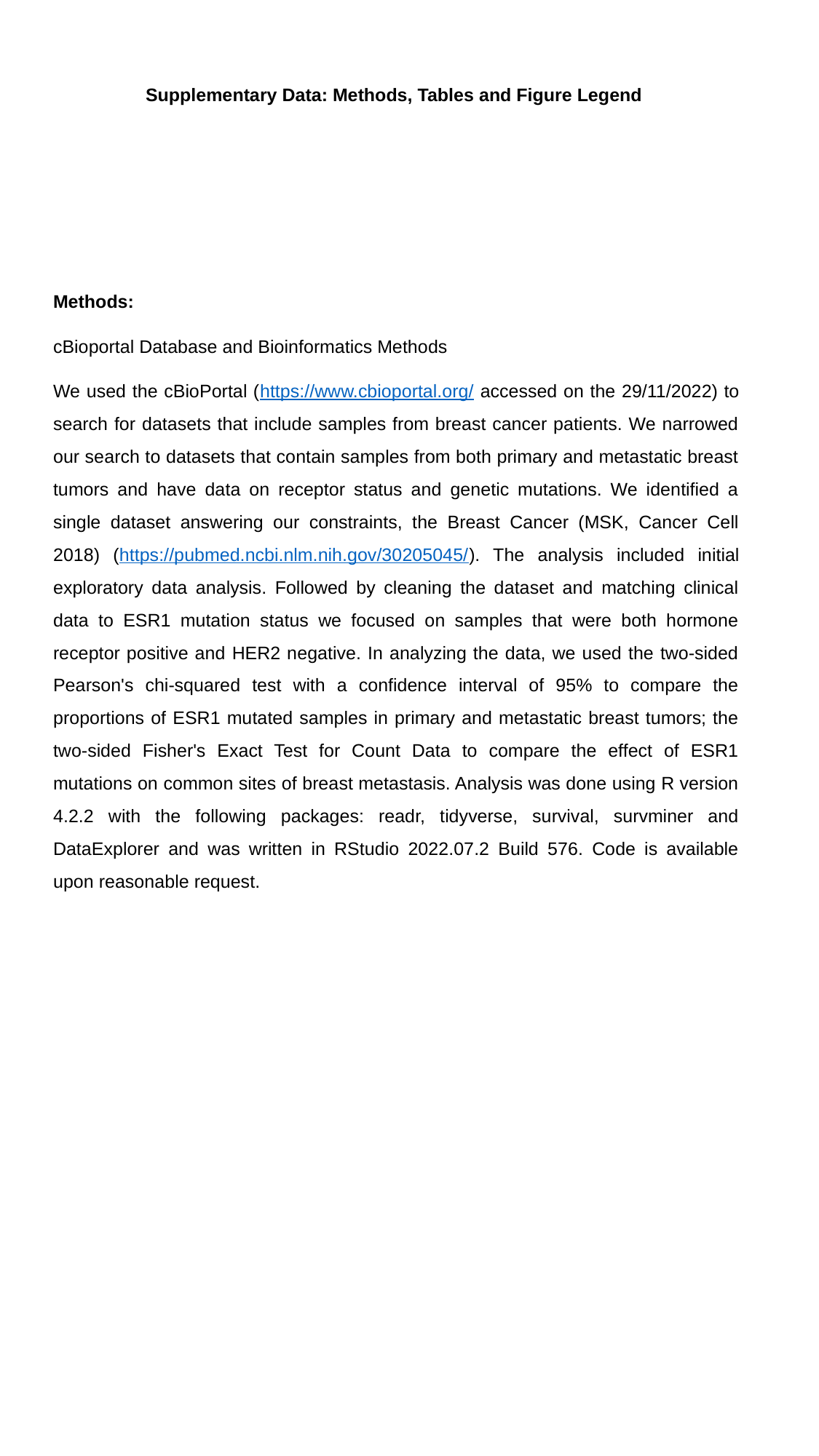

Supplementary Data: Methods, Tables and Figure Legend
Methods:
cBioportal Database and Bioinformatics Methods
We used the cBioPortal (https://www.cbioportal.org/ accessed on the 29/11/2022) to search for datasets that include samples from breast cancer patients. We narrowed our search to datasets that contain samples from both primary and metastatic breast tumors and have data on receptor status and genetic mutations. We identified a single dataset answering our constraints, the Breast Cancer (MSK, Cancer Cell 2018) (https://pubmed.ncbi.nlm.nih.gov/30205045/). The analysis included initial exploratory data analysis. Followed by cleaning the dataset and matching clinical data to ESR1 mutation status we focused on samples that were both hormone receptor positive and HER2 negative. In analyzing the data, we used the two-sided Pearson's chi-squared test with a confidence interval of 95% to compare the proportions of ESR1 mutated samples in primary and metastatic breast tumors; the two-sided Fisher's Exact Test for Count Data to compare the effect of ESR1 mutations on common sites of breast metastasis. Analysis was done using R version 4.2.2 with the following packages: readr, tidyverse, survival, survminer and DataExplorer and was written in RStudio 2022.07.2 Build 576. Code is available upon reasonable request.

## Slide 2
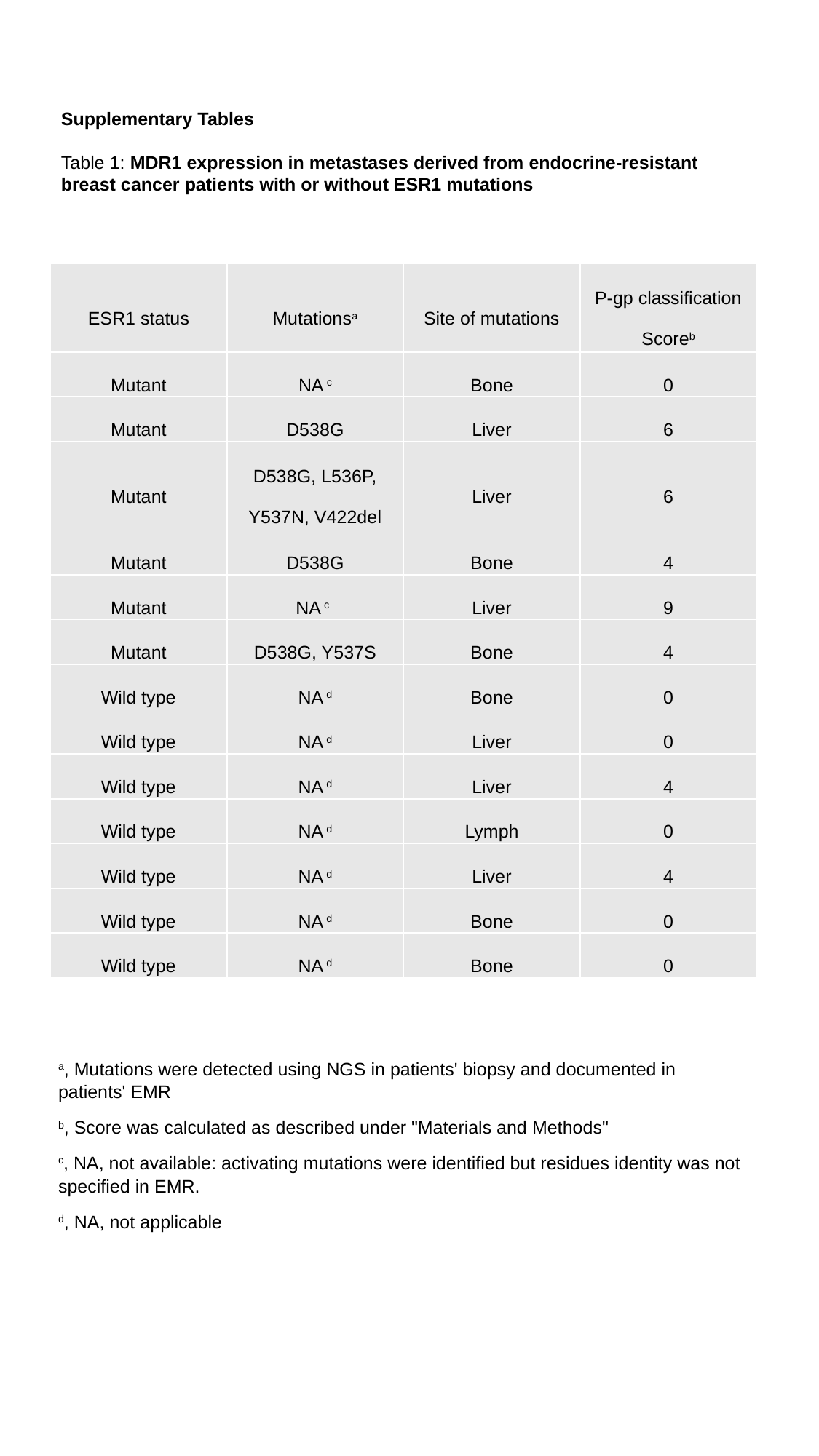

Supplementary Tables
Table 1: MDR1 expression in metastases derived from endocrine-resistant breast cancer patients with or without ESR1 mutations
| ESR1 status | Mutationsa | Site of mutations | P-gp classification Scoreb |
| --- | --- | --- | --- |
| Mutant | NA c | Bone | 0 |
| Mutant | D538G | Liver | 6 |
| Mutant | D538G, L536P, Y537N, V422del | Liver | 6 |
| Mutant | D538G | Bone | 4 |
| Mutant | NA c | Liver | 9 |
| Mutant | D538G, Y537S | Bone | 4 |
| Wild type | NA d | Bone | 0 |
| Wild type | NA d | Liver | 0 |
| Wild type | NA d | Liver | 4 |
| Wild type | NA d | Lymph | 0 |
| Wild type | NA d | Liver | 4 |
| Wild type | NA d | Bone | 0 |
| Wild type | NA d | Bone | 0 |
a, Mutations were detected using NGS in patients' biopsy and documented in patients' EMR
b, Score was calculated as described under "Materials and Methods"
c, NA, not available: activating mutations were identified but residues identity was not specified in EMR.
d, NA, not applicable
2

## Slide 3
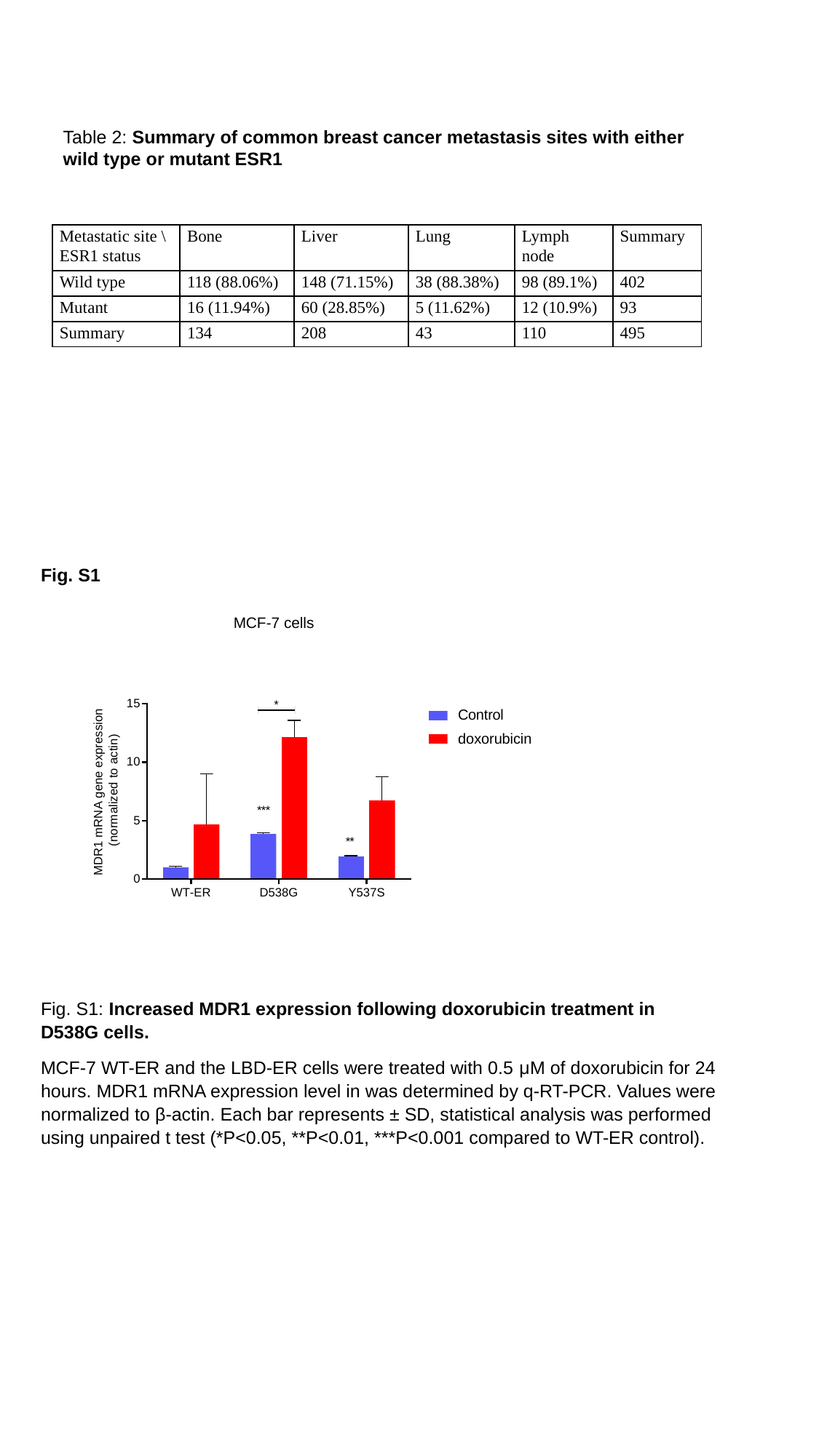

Table 2: Summary of common breast cancer metastasis sites with either wild type or mutant ESR1
| Metastatic site \ ESR1 status | Bone | Liver | Lung | Lymph node | Summary |
| --- | --- | --- | --- | --- | --- |
| Wild type | 118 (88.06%) | 148 (71.15%) | 38 (88.38%) | 98 (89.1%) | 402 |
| Mutant | 16 (11.94%) | 60 (28.85%) | 5 (11.62%) | 12 (10.9%) | 93 |
| Summary | 134 | 208 | 43 | 110 | 495 |
Fig. S1
MCF-7 cells
Fig. S1: Increased MDR1 expression following doxorubicin treatment in D538G cells.
MCF-7 WT-ER and the LBD-ER cells were treated with 0.5 μM of doxorubicin for 24 hours. MDR1 mRNA expression level in was determined by q-RT-PCR. Values were normalized to β-actin. Each bar represents ± SD, statistical analysis was performed using unpaired t test (*P<0.05, **P<0.01, ***P<0.001 compared to WT-ER control).

## Slide 4
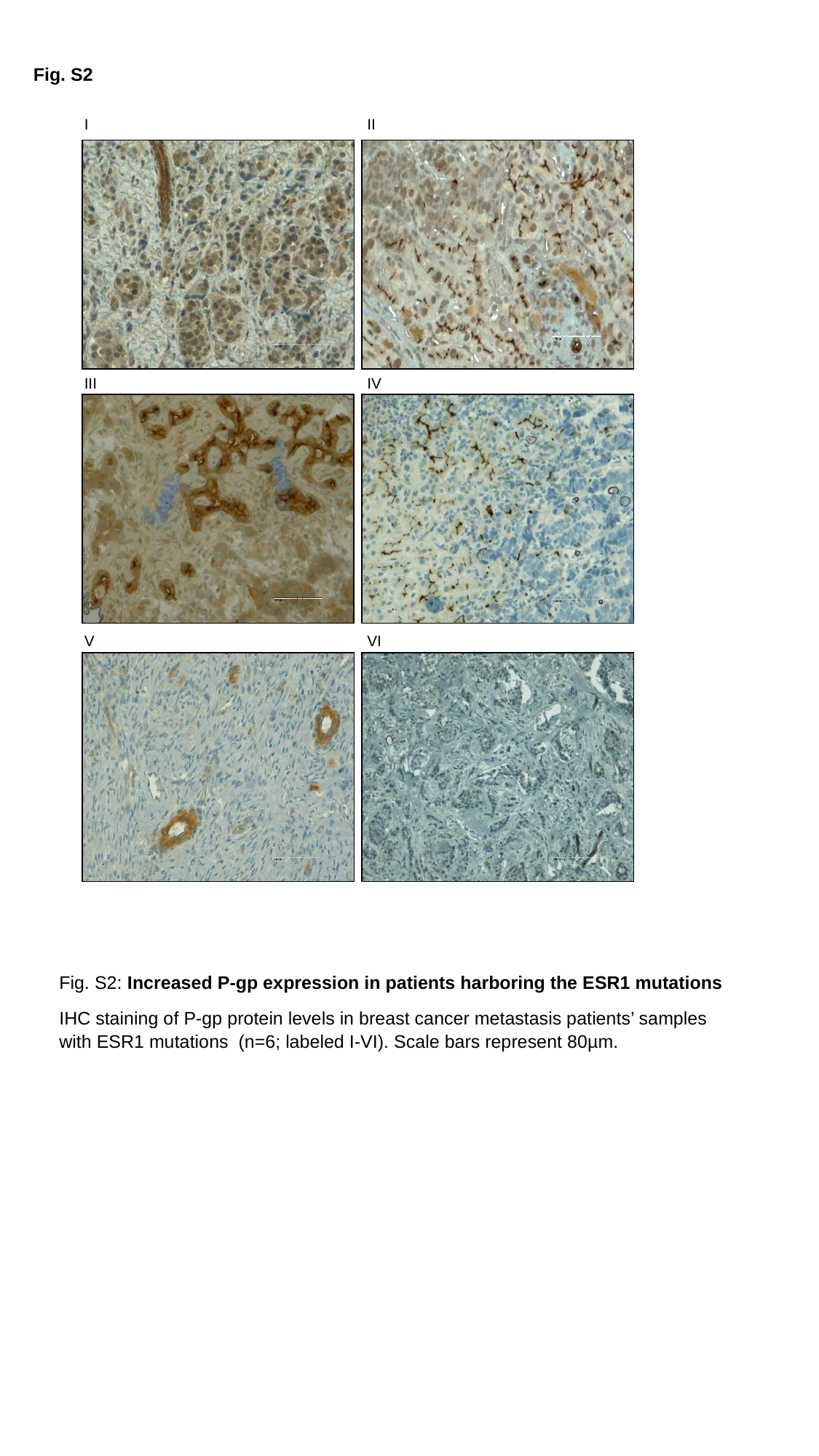

Fig. S2
I
II
III
IV
V
VI
Fig. S2: Increased P-gp expression in patients harboring the ESR1 mutations
IHC staining of P-gp protein levels in breast cancer metastasis patients’ samples with ESR1 mutations (n=6; labeled I-VI). Scale bars represent 80µm.

## Slide 5
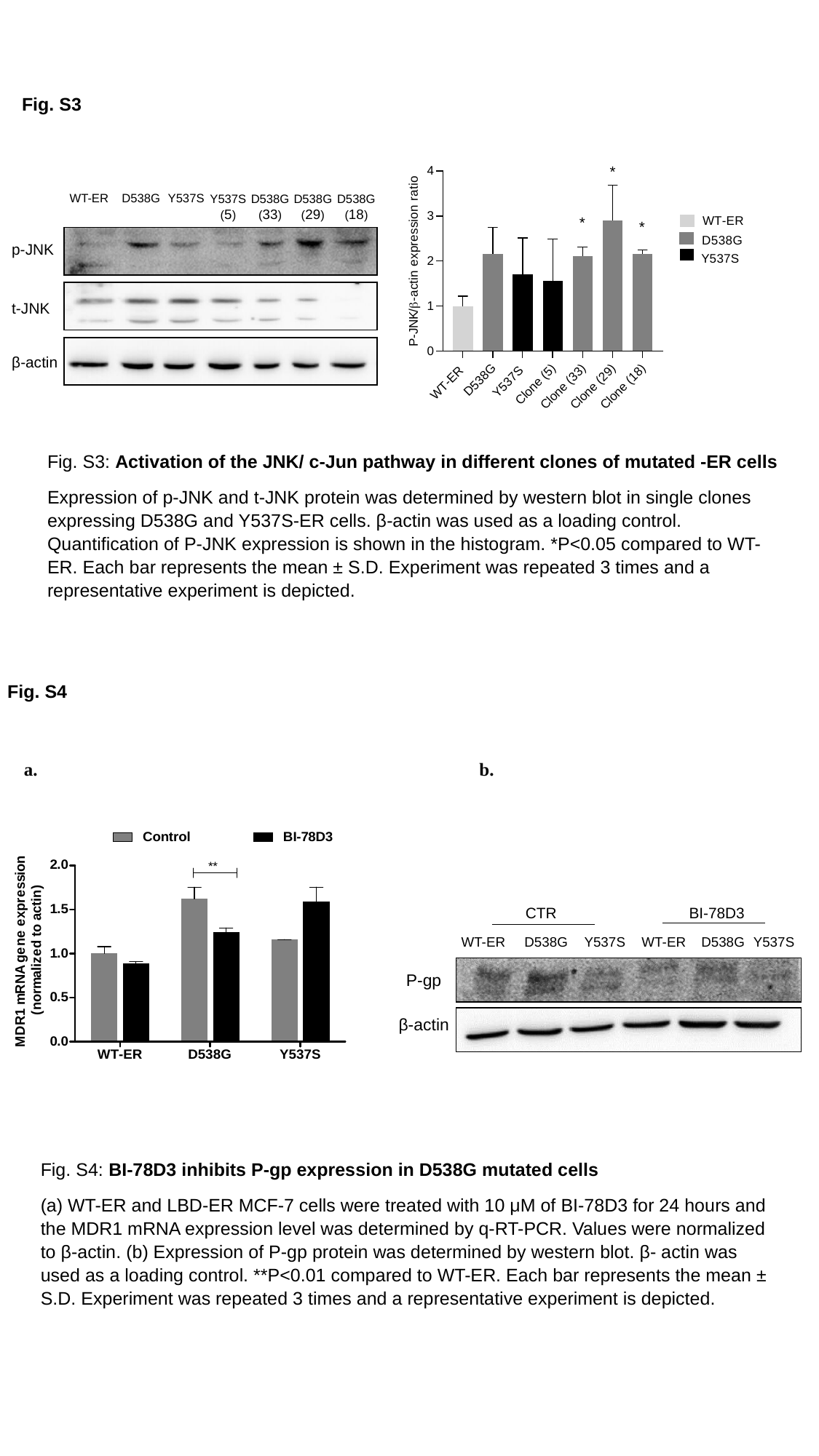

Fig. S3
WT-ER
D538G
Y537S
Y537S
(5)
D538G
(33)
D538G
(29)
D538G
(18)
p-JNK
t-JNK
β-actin
Fig. S3: Activation of the JNK/ c-Jun pathway in different clones of mutated -ER cells
Expression of p-JNK and t-JNK protein was determined by western blot in single clones expressing D538G and Y537S-ER cells. β-actin was used as a loading control. Quantification of P-JNK expression is shown in the histogram. *P<0.05 compared to WT-ER. Each bar represents the mean ± S.D. Experiment was repeated 3 times and a representative experiment is depicted.
Fig. S4
a.
b.
CTR
BI-78D3
WT-ER
D538G
Y537S
WT-ER
D538G
Y537S
P-gp
β-actin
Fig. S4: BI-78D3 inhibits P-gp expression in D538G mutated cells
(a) WT-ER and LBD-ER MCF-7 cells were treated with 10 μM of BI-78D3 for 24 hours and the MDR1 mRNA expression level was determined by q-RT-PCR. Values were normalized to β-actin. (b) Expression of P-gp protein was determined by western blot. β- actin was used as a loading control. **P<0.01 compared to WT-ER. Each bar represents the mean ± S.D. Experiment was repeated 3 times and a representative experiment is depicted.

## Slide 6
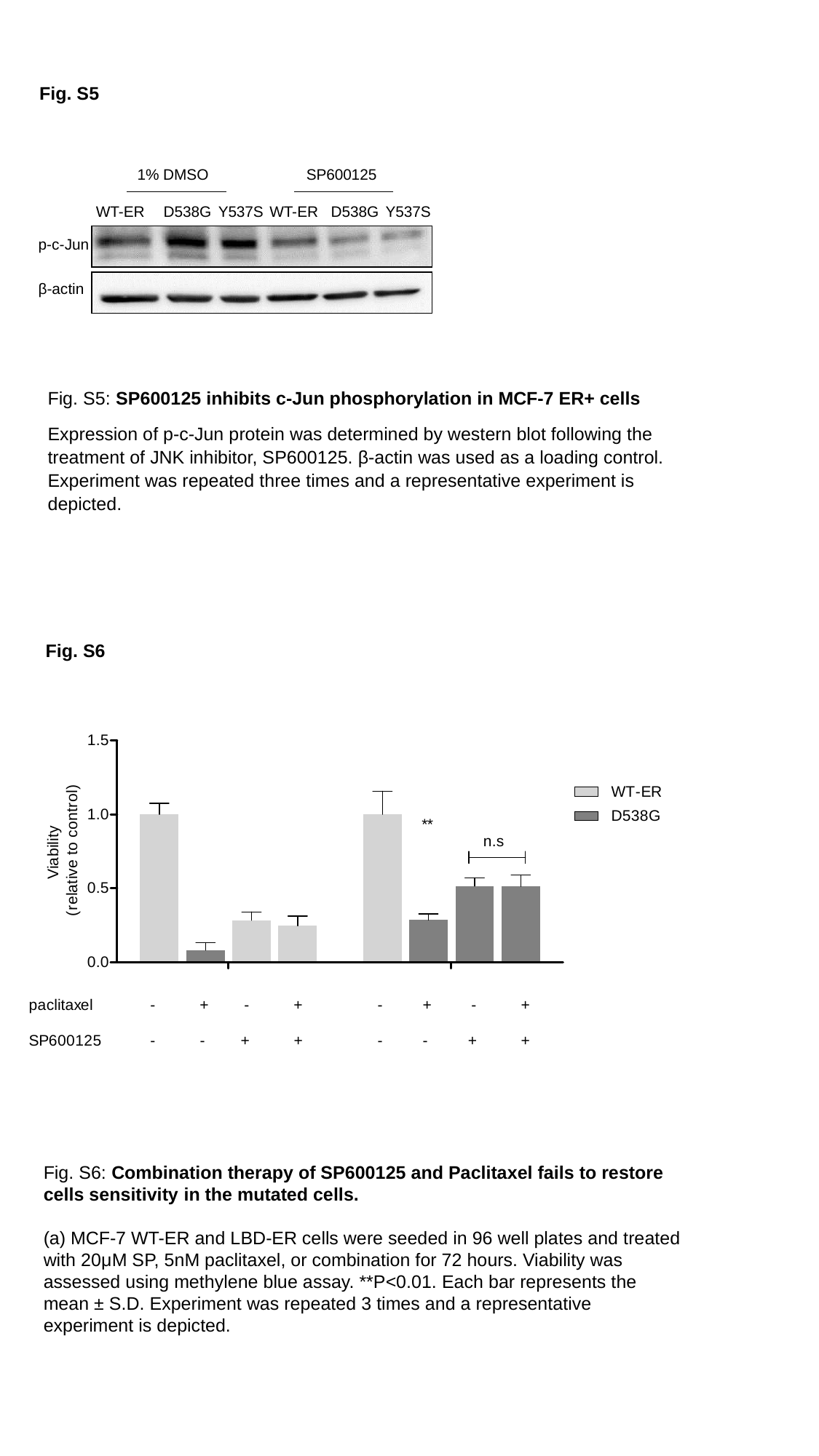

Fig. S5
1% DMSO
SP600125
WT-ER
D538G
Y537S
WT-ER
D538G
Y537S
p-c-Jun
β-actin
Fig. S5: SP600125 inhibits c-Jun phosphorylation in MCF-7 ER+ cells
Expression of p-c-Jun protein was determined by western blot following the treatment of JNK inhibitor, SP600125. β-actin was used as a loading control. Experiment was repeated three times and a representative experiment is depicted.
Fig. S6
Fig. S6: Combination therapy of SP600125 and Paclitaxel fails to restore cells sensitivity in the mutated cells.
(a) MCF-7 WT-ER and LBD-ER cells were seeded in 96 well plates and treated with 20μM SP, 5nM paclitaxel, or combination for 72 hours. Viability was assessed using methylene blue assay. **P<0.01. Each bar represents the mean ± S.D. Experiment was repeated 3 times and a representative experiment is depicted.
